# A Rare Presentation of IgA Nephropathy in a 62-Year-Old Hispanic Female

**DOI:** 10.7759/cureus.38783

**Published:** 2023-05-09

**Authors:** Sarah C Kurkowski, Michael J Thimmesch, Osama Elkhider, Yasir Abdelgadir

**Affiliations:** 1 Medical School, Medical College of Wisconsin, Milwaukee, USA; 2 Department of Medicine, Medical College of Wisconsin, Milwaukee, USA

**Keywords:** mestc score, diabetic kidney disease, hypertension, diabetes, end-stage renal disease, chronic kidney disease, iga nephropathy

## Abstract

A case of immunoglobulin A (IgA) nephropathy is presented here that demonstrates an unusual clinical presentation in multiple ways and is vitally important for clinicians to consider. The patient is a Hispanic female in her 7th decade of life that presented with nephrotic-range proteinuria without hematuria ultimately leading to a diagnosis of IgA nephropathy. After diagnosis, her clinical course was complicated by continued poorly controlled type II diabetes mellitus and hypertension, and ultimately her kidney disease progressed to chronic kidney disease IV and then end-stage renal disease requiring hemodialysis. Though IgA nephropathy predominantly presents as nephritic syndrome, it can also present as nephrotic range proteinuria and even rapidly progressive glomerulonephritis which should be considered even when the patient's ethnicity and age group carry a smaller risk.

## Introduction

Immunoglobulin A (IgA) nephropathy is an immune complex disease in which IgA antigen-antibody complexes are deposited in the mesangium. It is the most common glomerulonephritis (GN); however, its prevalence is less than diabetic kidney disease and hypertensive kidney disease [1].

The hallmark of IgA nephropathy diagnosis is the predominance of IgA deposits within the glomerular mesangium. The deposits can be primarily composed of solely IgA or IgA in combination with IgG, IgM, or complement C3 [2]. The disease pathway has long been studied to better understand what prompts the immune system to begin attacking the glomerular mesangium and depositing IgA. Genome-wide association studies have shown that a patient's susceptibility to the disease is affected by several common variants in the antigen processing and presentation pathway [3]. The leading hypothesis is that the body’s immune system is activated due to underglycosylation in the hinge region of IgA1 which leads to the development of antibodies and immune complexes against the IgA1 (which is deficient in galactose glycans). The glomerular deposits of immune complexes, which contain IgA1, activate the mesangial cells within the glomerulus. This leads to the overproduction of cytokines and chemokines and the activation of the complement pathway, ultimately leading to tissue injuries, such as podocyte and tubulointerstitial injury [2,4].

Interestingly, there is a relationship between patient ethnicity, age, and sex on the risk of developing IgA nephropathy. The disease is more common in Caucasian and Asian patients and, in the United States, affects males more than females in a 2:1 ratio. In one study of patients with IgA nephropathy, 60% were male, 38% were Caucasian, 36% were Asian/Pacific Islander, 19% were Hispanic, 3% were African American, and 4% were of another unknown ethnicity [5]. The first sign of kidney disease is usually found within the second to fourth decade of life. Therefore, IgA nephropathy can affect patients of all ages but is most common between the ages of 10 and 39 years old [1,6].

The clinical presentation of IgA nephropathy can differ greatly between individual patients. The most common presentation is recurrent episodes of gross hematuria after an upper respiratory tract infection or gastroenteritis (about 50% of patients). About 33% of patients present with microscopic hematuria and mild proteinuria [7]. Overall, the most dominant presenting symptom is hematuria, whether gross or microscopic. IgA nephropathy in children more commonly presents with macroscopic hematuria and follows an upper respiratory tract infection, which is considered the “classic clinical syndrome.” However, this classic presentation only occurs in about 10-15% of adult patients [8,9]. Within the adult population, the disease most commonly presents with microscopic hematuria and varying degrees of proteinuria (ranging from mild to nephrotic syndrome), with or without the presence of kidney disease. Nephrotic syndrome on presentation is rare but, if present, has dire consequences such as infection, hypocalcemia (and thus leading to bony abnormalities), hyperlipidemia (increased risk for atherosclerosis), hypercoagulability, and hypovolemia. Some patients present with nephrotic range proteinuria, less severe than nephrotic syndrome, though they can develop nephrotic syndrome later on in the clinical course [10].

Approximately 20-40% of patients develop end-stage renal disease (ESRD) in 10-30 years from IgA nephropathy diagnosis or the first clinical presentation of the disease [4,5]. The disease progression varies widely between patients with some never progressing to ESRD and even ones who have a spontaneous remission of the disease [8].

## Case presentation

The patient in this case is a 62-year-old Hispanic female with a past medical history of poorly controlled hypertension, poorly controlled type II diabetes mellitus, and heart failure with preserved ejection fraction. She presented to the emergency department with three days of a worsening headache, nausea, vomiting, and uncontrolled hypertension, along with two days of diarrhea. For the three days prior to admission, she was unable to tolerate oral intake or medications. She endorsed mild dyspnea and sharp, mid-sternal chest pain, both of which were resolved in the emergency department. Her blood pressure at admission was as high as 251/126. Electrocardiogram was unremarkable, showing normal sinus rhythm. CT head was negative for acute hemorrhage, mass effect, or trauma. The infectious workup was negative. On admission labs, the patient was found to have an acute kidney injury (AKI). Her serum creatinine for the year prior to admission ranged from 0.98 to 1.38, rising to 2.64 on admission. In addition, the basic metabolic panel was notable for hyperkalemia, hyperchloremia, low bicarbonate, high blood urea nitrogen, and low estimated glomerular filtration rate (Table 1). Elevated blood pressure was treated initially with intravenous hydralazine, and the patient was admitted to the medicine team for inpatient management.

**Table 1:** Laboratory values on admission FeNa: fractional excretion of sodium (Na), eGFR: estimated glomerular filtration rate, BUN: blood urea nitrogen

Lab	Na (mmol/L)	K (mmol/L)	Cl (mmol/L)	HCO3 (mmol/L)	BUN (mg/dL)	Cr (mg/dL)	eGFR (mL/min/1.73m²)	Urine Na (mmol/L)	Urine Cr (mmol/L)	FeNa	Urine random protein
Lab value	140	5.4	111	18	40	2.64 prior to admission baseline of 1.1-1.3	20	67	55	2.3%	1058
Normal range	136-145	3.4-5.1	96-105	22-29	6-23	0.50-1.10	≥ 60			> 2% = intrinsic process	

Upon admission, a renal ultrasound was performed and demonstrated a distended bladder with no hydronephrosis, and a bladder scan showed urinary retention. A urinary catheter was placed to treat the urinary retention. The fractional excretion of sodium (FeNa) was consistent with an intrinsic renal process leading to the AKI. Twenty-four-hour urine protein was elevated at 6.7 grams. Of note, the patient had no hematuria (gross or microscopic) neither prior to admission nor upon admission's urinalysis. For at least the year prior to admission, she followed regularly with a primary care provider. The most recent urinalysis before admission was 10 months prior which was notable for urine protein of 200 mg/dL (reference range of <20 mg/dL), while her urinalysis on admission had urine protein of 300 mg/dL. The patient's complement levels were normal, and ANA, ANCA, anti-double-stranded DNA, and anti-PLA2R IgG antibodies were negative (Table 2).

**Table 2:** Autoimmune panel C3: complement component 3, C4: complement component 4, ANA: antinuclear antibody, ANCA IFA: antineutrophil cytoplasmic antibody indirect fluorescent antibody, anti-PLA2R IgG: anti-phospholipase A2 receptor immunoglobulin G antibodies

Lab	C3 (mg/dL)	C4 (mg/dL)	ANA	ANCA IFA titer	Anti-double-stranded DNA	Anti-PLA2R IgG
Lab value	102	45	None detected	<1:20	None detected	<1:10
Normal range	90-180	10-40	None detected	<1:20	None detected	<1:10
Clinical interpretation	Negative	Mild elevation	Negative	Negative	Negative	Negative

Urinary retention was resolved, and the urinary catheter was removed on admission day 7. A renal biopsy was performed on admission day 10. Biopsy results demonstrated IgA-dominant GN versus nephropathy, nodular diabetic nephropathy with focal global and focal segmental glomerular sclerosis (45% and 40%), severe interstitial fibrosis and tubular atrophy (~50%), severe arteriosclerosis, and severe arteriolar hyalinosis. Between IgA-dominant GN and IgA nephropathy, IgA nephropathy was favored because of the absence of known infectious history, mesangial localization of deposits without subepithelial hump deposits, and lack of prominent neutrophilic infiltration within glomerular capillaries. Using the Oxford Classification of IgA nephropathy, the patient’s biopsy showed mesangial hypercellularity (M1), segmental sclerosis (S1), and severe interstitial fibrosis and tubular atrophy (T2) without endocapillary hypercellularity (E0) or crescents (C0). As the IgA nephropathy is overlaying a background of very severe diabetic and hypertensive nephropathy, the M1/S1/T2 could also be attributed to the diabetic nephropathy.

On discharge, eleven days post-admission, the patient’s hyperkalemia, hyperchloremia, hypertension, and urinary retention were resolved. Bicarbonate levels still remained low, and BUN was still elevated (increased from levels at admission) (Table 3). Serum creatinine over the hospital stay had risen to and plateaued at ~3.4 mg/dL without the need for renal replacement therapy. The patient was discharged with close nephrology follow-up, bumetanide for extremity edema, and losartan for proteinuria.

**Table 3:** Laboratory values at discharge FeNa: fractional excretion of sodium (Na), eGFR: estimated glomerular filtration rate, BUN: blood urea nitrogen

Lab	Na (mmol/L)	K (mmol/L)	Cl (mmol/L)	HCO3 (mmol/L)	BUN (mg/dL)	Cr (mg/dL)	eGFR (mL/min/1.73m²)
Lab value	137	4.1	108	18	63	3.39	15
Normal range	136-145	3.4-5.1	96-105	22-29	6-23	0.50-1.10	≥ 60

On nephrology follow-up, 10 days post-discharge, the renal biopsy results were discussed with the patient, and it was determined she had chronic kidney disease stage IV (CKDIV). The patient was also complaining of hallucinations, dizziness, and worsening leg edema since discharge. It was determined that she was a poor candidate for immunosuppressive therapy due to her severe CKD and poor control of hypertension and type II diabetes mellitus. At this follow-up, her serum creatine levels remained stable post-discharge, and her new baseline creatine level was determined to be 3.1-3.3.

Over the next two months post-discharge, the patient continued to follow with nephrology as well as urology. She was started on sodium zirconium cyclosilicate outpatient due to worsening hyperkalemia; however, the patient was not compliant. She was admitted to the hospital twice during this time. One admission for hyperkalemia and volume overload (presumed heart failure exacerbation on top of CKD), though the patient left against medical advice prior to treatment. The most recent admission was for altered mental status and edema. She was found to have progressed to ESRD, and hemodialysis was started. Her mentation dramatically improved with the initiation of hemodialysis, and it was determined she will require life-long hemodialysis.

## Discussion

This patient case presentation is remarkable for several reasons. First, our patient is Hispanic, which is a population that less commonly suffers from IgA nephropathy when compared to Caucasian and Asian/Pacific Islander populations. As stated prior, one study that looked at clinical and pathological data of 298 patients with primary glomerular lesions found that 149/298 patients had IgA nephropathy. Of these 149 patients, the ethnic breakdown of the patients is as follows: 38% Caucasian, 36% Asian/Pacific Islander, 19% Hispanic, 3% African American, and 4% of another unknown ethnicity. The severity of IgA nephropathy did not differ based on the race or ethnicity of the patient [5]. In addition, our patient is a female, whereas IgA nephropathy in the United States affects males more than females at a 2:1 ratio [5,6]. The fact that our patient is a Hispanic female makes her presentation of IgA nephropathy much less common than the usual patient presentation.

Second, the case presented here is unique as the patient presented clinically for the first time in her seventh decade of life. This is an unusual presentation of IgA nephropathy. There is of course the option that she was asymptomatic prior to admission and so did not seek medical care. Though for at least the year prior to admission, she was following up regularly with a primary care provider.

Third, the clinical presentation of our patient included nephrotic-range proteinuria without hematuria (microscopic or macroscopic), which is quite uncommon. For explanation purposes, nephrotic-range proteinuria is defined as “the urinary loss of 3 grams or more of protein per 24 hours or, on a single spot urine sample, the presence of 2 grams of protein per gram of urinary creatinine” [10]. On a urine dipstick, it will produce readings of 3+ or 4+ protein. Urine samples should be taken over 24 hours to be accurate, as done in our patient’s workup. Nephrotic-range proteinuria must be differentiated from nephrotic syndrome, as the two can be confused though quite different. Nephrotic syndrome is massive proteinuria (>3.5g/d) leading to hypoalbuminemia (< 30g/L), hyperlipidemia, edema, hypercoagulability, and other resulting complications [10]. Our patient did not have nephrotic syndrome; however, nephrotic-range proteinuria does have the potential to progress to nephrotic syndrome. Our patient’s lack of hematuria on clinical presentation is uncommon. There are multiple known presentations of IgA nephropathy: synpharyngitic macroscopic hematuria, postpharyngitic macroscopic hematuria, rapidly progressive glomerulonephritis (RPGN), and nephrotic syndrome (as discussed above). The classic clinical syndrome is synpharyngitic macroscopic hematuria, which is when episodes of gross/macroscopic hematuria occur concurrently within two to three days after the onset of infection. However, this is much more common in the pediatric population compared to the adult population (only 10-15% of adults present with this clinical picture and are usually < 40 years old). This presentation has a more favorable prognosis [9]. Our patient presented here did not present with the classic presentation, though that would be expected as she is in an older age category (> 40 years old). What makes her presentation quite rare is the absence of hematuria, both micro and macroscopic. Recurrent episodes of visible hematuria after an upper respiratory tract infection or gastroenteritis are seen in approximately 50% of patients and another 33% of patients present with microscopic hematuria and mild proteinuria. The absence of hematuria puts our patient’s case in the remaining 17% of patients [7].

Of note, 44% of patients diagnosed with IgA nephropathy had nephrotic-range proteinuria at the time of kidney biopsy [5] as did our patient. Interestingly, on our patient’s final kidney biopsy report, it was stated that the IgA deposits could be from two entities: IgA-dominant GN (meaning secondary IgA GN) versus IgA nephropathy. IgA nephropathy was favored because of the absence of known infectious history, mesangial localization of deposits without subepithelial hump deposits, and lack of prominent neutrophilic infiltration within glomerular capillaries [11]. IgA-dominant GN is a specific form of postinfectious GN that demonstrates IgA-dominant deposits, thereby mimicking IgA nephropathy. However, this disease pattern is most often seen in diabetic patients with known staphylococcal infections [12].

Ultimately, our patient was treated with a loop diuretic bumetanide and (angiotensin receptor blocker (ARB)) losartan. The backbone of therapy for IgA nephropathy is proteinuria and blood pressure and control with either an angiotensin-converting enzyme inhibitor or ARB. In patients with a high risk of chronic kidney disease progression, corticosteroids can be considered. Newly approved medications for IgA nephropathy are budesonide (a glucocorticoid) and sparsentan. Sparsentan is an antagonist of both the endothelin type A receptor and the angiotensin II (Ang II) type 1 receptor, thereby blocking the action of Ang II and endothelin-1. This reduces the vasoconstriction and mitogenic actions of the agents. Immunosuppression can also be considered, but studies have shown variable effects on renal function preservation and proteinuria reduction [8]. For our patient, corticosteroid therapy and immunosuppression were not used due to her severe CKD and poor control of hypertension and type II diabetes mellitus. Another treatment option is kidney transplantation, especially for patients with ESRD. However, the literature states that even after a kidney transplant, IgA nephropathy recurs in up to 50% of patients with renal allograft [13].

There are multiple prognostic indicators of IgA nephropathy. Clinical features that are strong predictors of the risk of progressing to chronic kidney disease are heavy proteinuria at presentation or during follow-up, elevated serum creatinine at presentation, and hypertension at presentation. Our patient presented with each of these clinical features, indicating that her risk of progressing to chronic kidney disease was high (and she ultimately did progress to CKDIV and then ESRD requiring long-term hemodialysis). Weaker predictors of poor prognosis are male sex, older age at presentation, and absence of recurrent macroscopic hematuria [2]. Again, our patient was in her 7th decade of life (older age) and had no history of macroscopic hematuria on presentation. It is possible that with her history of diabetes, the renal disease is not entirely due to IgA nephropathy but is a combination of both diabetic kidney disease and IgA nephropathy. A systematic review and meta-analysis of studies assessing the Oxford classification of IgA nephropathy from January 2009 to December 2012 showed that M (mesangial hypercellularity), S (segmental glomerulosclerosis), T (tubular atrophy/interstitial fibrosis), and C (crescent) lesions were strongly associated with the progression to kidney failure. However, E (endocapillary hypercellularity) lesions were not strongly associated. This review and meta-analysis included 16 retrospective cohort studies with 3,893 patients. The authors used a multivariate model to calculate hazard ratios for kidney failure. The highest hazard ratio (3.2, 95% confidence interval) was found with T1/2 lesions (> 25% tubular atrophy or interstitial fibrosis). The hazard ratios of 0.6, 1.8, and 2.3 were for scores of M0 (mesangial hypercellularity score ≤ 0.5), S1 (presence of segmental glomerulosclerosis), and C (crescent) lesions, respectively [14]. Our patient’s biopsy had a MEST-C pathological score of M1/E0/S1/T2/C0, adding yet again multiple other indicators that her IgA nephropathy would progress to poor clinical outcomes [2,14].

## Conclusions

In conclusion, the case of IgA nephropathy presented here is unique in multiple regards. The patient was a Hispanic female in her 7th decade of life that presented with AKI nephrotic-range proteinuria without hematuria ultimately leading to a diagnosis of IgA nephropathy. There is a possibility that she was asymptomatic for a period of time prior to presentation, though she was following up regularly with a primary care provider, and the previous urinalysis did not demonstrate hematuria. In addition, her history of diabetes could be contributing to the severity of the disease on presentation and may represent a combination of diabetic kidney disease and IgA nephropathy. As detailed previously, her age, race, and absence of hematuria on presentation are all individually rare characteristics but all together make it an interesting presentation.
